# A swine arterivirus deubiquitinase stabilizes two major envelope proteins and promotes production of viral progeny

**DOI:** 10.1371/journal.ppat.1009403

**Published:** 2021-03-18

**Authors:** Rui Guo, Xingyu Yan, Yanhua Li, Jin Cui, Saurav Misra, Andrew E. Firth, Eric J. Snijder, Ying Fang

**Affiliations:** 1 Department of Diagnostic Medicine and Pathobiology, College of Veterinary Medicine, Kansas State University, Manhattan, Kansas, United States America; 2 Department of Pathobiology, College of Veterinary Medicine, University of Illinois, Urbana-Champaign, Urbana, Illinois, United States America; 3 Department of Biochemistry and Molecular Biophysics, College of Arts and Sciences, Kansas State University, Manhattan, Kansas, United States America; 4 Department of Pathology, University of Cambridge, Cambridge, United Kingdom; 5 Department of Medical Microbiology, Leiden University Medical Center, Leiden, The Netherlands; University of Kansas, UNITED STATES

## Abstract

Arteriviruses are enveloped positive-strand RNA viruses that assemble and egress using the host cell’s exocytic pathway. In previous studies, we demonstrated that most arteriviruses use a unique -2 ribosomal frameshifting mechanism to produce a C-terminally modified variant of their nonstructural protein 2 (nsp2). Like full-length nsp2, the N-terminal domain of this frameshift product, nsp2TF, contains a papain-like protease (PLP2) that has deubiquitinating (DUB) activity, in addition to its role in proteolytic processing of replicase polyproteins. In cells infected with porcine reproductive and respiratory syndrome virus (PRRSV), nsp2TF localizes to compartments of the exocytic pathway, specifically endoplasmic reticulum-Golgi intermediate compartment (ERGIC) and Golgi complex. Here, we show that nsp2TF interacts with the two major viral envelope proteins, the GP5 glycoprotein and membrane (M) protein, which drive the key process of arterivirus assembly and budding. The PRRSV GP5 and M proteins were found to be poly-ubiquitinated, both in an expression system and in cells infected with an nsp2TF-deficient mutant virus. In contrast, ubiquitinated GP5 and M proteins did not accumulate in cells infected with the wild-type, nsp2TF-expressing virus. Further analysis implicated the DUB activity of the nsp2TF PLP2 domain in deconjugation of ubiquitin from GP5/M proteins, thus antagonizing proteasomal degradation of these key viral structural proteins. Our findings suggest that nsp2TF is targeted to the exocytic pathway to reduce proteasome-driven turnover of GP5/M proteins, thus promoting the formation of GP5-M dimers that are critical for arterivirus assembly.

## Author summary

Arteriviruses are a rapidly expanding family of positive-stranded RNA viruses, which includes economically important veterinary pathogens like equine arteritis virus (EAV) and two species of porcine reproductive and respiratory syndrome virus (PRRSV-1 and PRRSV-2). In our previous studies, we uncovered an unprecedented arterivirus gene expression mechanism: a highly efficient -2 programmed ribosomal frameshift (PRF) that is controlled by an interaction of viral protein nsp1ß with specific RNA sequences and host poly(C) binding proteins. It is used by PRRSVs, and most other arteriviruses, to efficiently produce a previously unknown nonstructural protein variant, nsp2TF. In this study, we demonstrate that PRRSV nsp2TF interacts with the two major arteriviral envelope proteins, GP5 and M, whose heterodimerization in the secretory pathway is a key step in envelope protein trafficking and virus assembly. Our findings suggest that nsp2TF promotes arterivirus assembly by antagonizing the ubiquitination-dependent proteasomal degradation of GP5 and M proteins. This mechanism is based on the DUB activity of the PLP2 protease domain located within the N-terminal region of nsp2TF. To our knowledge, this is the first study to demonstrate that viruses can express a DUB that functions specifically to counteract the ubiquitination and degradation of key viral structural proteins.

## Introduction

Porcine reproductive and respiratory syndrome virus (PRRSV) belongs to the order *Nidovirales*, family *Arteriviridae*. Arteriviruses have an intriguing distant evolutionary relationship to coronaviruses and other members of the order *Nidovirales*, but possess many characteristics unique among currently known positive-stranded RNA viruses (reviewed in [1]). Besides PRRSV, the arterivirus family includes equine arteritis virus (EAV), mouse lactate dehydrogenase elevating virus (LDV), simian hemorrhagic fever virus (SHFV) and a variety of more recently identified members, many of which are of simian origin [2,3].

PRRSV is a small, enveloped virus, with a ~15 kb genome that contains 11 known open reading frames (ORFs). The 3’-proximal quarter of the viral genome encodes four envelope glycoproteins (GP2a, GP3, GP4 and GP5), three non-glycosylated trans-membrane proteins (E, ORF5a, and M) and the nucleocapsid protein (N). The viral structural proteins are expressed upon synthesis of a nested set of subgenomic mRNAs. Arteriviral envelope proteins are co-translationally inserted into the endoplasmic reticulum (ER) membrane and then travel through the host cell secretory pathway. While trafficking through the pathway, the viral envelope proteins engage in virus assembly by interacting with each other and with viral nucleocapsids [1]. Based on electron microscopy studies, arterivirus budding is thought to occur from ‘smooth intracellular membranes’, but the identity of the compartment(s) involved has not been studied in detail. In terms of sequence conservation, glycoprotein GP5 is the most variable structural protein, in particular its N-terminal ectodomain. The protein also contains a central triple-spanning transmembrane region and a cytoplasmic C-terminal domain [4]. An N-terminal signal peptide is predicted to be cleaved from the GP5 ectodomain [4] and the remainder of the N-terminal sequence is post-translationally modified with varying numbers of N-linked glycans during GP5 maturation in the ER and Golgi complex [5,6]. Studies of EAV and LDV have shown that GP5 and the non-glycosylated M protein, which also spans the membrane three times, form a disulfide-linked heterodimer [5–7]. Heterodimerization triggers a series of important events, including transport of the GP5-M complex from the ER through the ER-Golgi intermediate compartment (ERGIC) to the Golgi complex, where the maturation of the GP5-linked glycans occurs. Presumably, some or most of the GP5-M dimers have already been incorporated into new virions when they reach the Golgi complex [5,7].

The 5’ three-quarters of the PRRSV genome contain two large open reading frames, 1a and 1b, which encode two large nonstructural polyproteins, pp1a and pp1ab. Expression of pp1ab depends on a -1 programmed ribosomal frameshift (PRF) at the junction of ORF1a and ORF1b. The pp1a and pp1ab replicase polyproteins are processed into at least 14 nonstructural proteins (nsps), including nsp1α/β and nsp2-nsp12. These nsps presumably assemble into a membrane-associated replication and transcription complex (RTC) [1,8]. In our previous study, we discovered that PRRSV, and apparently most other arteriviruses, use an unusual -2 PRF mechanism to efficiently express an additional, previously unknown nonstructural protein, nsp2TF [9]. Ribosomal frameshifting accesses a trans-frame (TF) ORF that overlaps with the nsp2-coding region of ORF1a in the -2 frame. This PRF depends on the interaction of the viral protein nsp1β with specific RNA sequences near the PRF site and host poly(C) binding proteins [10,11]. The result is a transframe fusion protein consisting of the N-terminal two-thirds of nsp2 followed by a unique C-terminal domain that is specified by the TF ORF. Moreover, -1 PRF also occurs at the same PRF site. Due to the presence of a translation termination codon in the -1 reading frame immediately downstream, -1 PRF results in production of a truncated form of nsp2, termed “nsp2N” [9,10]. Specific inactivation of this newly discovered PRF mechanism results in PRRSV mutants that are viable but clearly crippled in terms of plaque size and infectious progeny titers [9,10]. The distinct C-terminal domains of nsp2 and nsp2TF appear to direct these proteins to different subcellular compartments [9]. Full-length nsp2 is a key player in the formation of the double-membrane replication organelles, with which arteriviral RNA synthesis is associated [12,13]. In contrast, nsp2TF is not targeted to replication organelles but instead localizes to compartments of the exocytic pathway, which is used for arterivirus assembly and egress [9].

All three nsp2 variants (nsp2, nsp2TF and nsp2N) include an N-terminal papain-like proteinase domain (PLP2) that directs the critical cleavage of the nsp2/3 site in the replicase polyproteins. In addition to its critical role in replicase polyprotein processing, arterivirus PLP2 was found to exhibit a DUB activity, which is thought to target cellular substrates, such as critical factors in innate immune response pathways that depend on ubiquitination for their function [14–18]. As an evasion strategy, viruses have developed various strategies to interfere with or reverse ubiquitination, including the expression of DUBs from the viral genome [18,19]. In a previous study, we demonstrated that expression of the PRRSV PLP2 domain could inhibit NF-kB activation by reducing polyubiquitination of IkB-α, thereby downregulating IkB-α degradation by the proteasome [14]. More recently, we confirmed that nsp2TF also functions as a DUB and antagonizes ubiquitination of host cell proteins [20].

In the present study, we demonstrate that PRRSV nsp2TF interacts with the two major arteriviral envelope proteins, GP5 and M, during their transport along the exocytic pathway. Moreover, we show that nsp2TF, through its DUB activity, down-regulates polyubiquitination and proteasomal degradation of GP5 and M proteins, thereby stabilizing these key viral structural components and promoting the production of viral progeny. Our study reveals a novel function of nsp2TF and provides new insights into the mechanisms of structural protein maturation and virus assembly in the PRRSV replication cycle.

## Results

### Nsp2TF co-localizes with GP5 and M protein in PRRSV-infected cells

**Fig 1:**
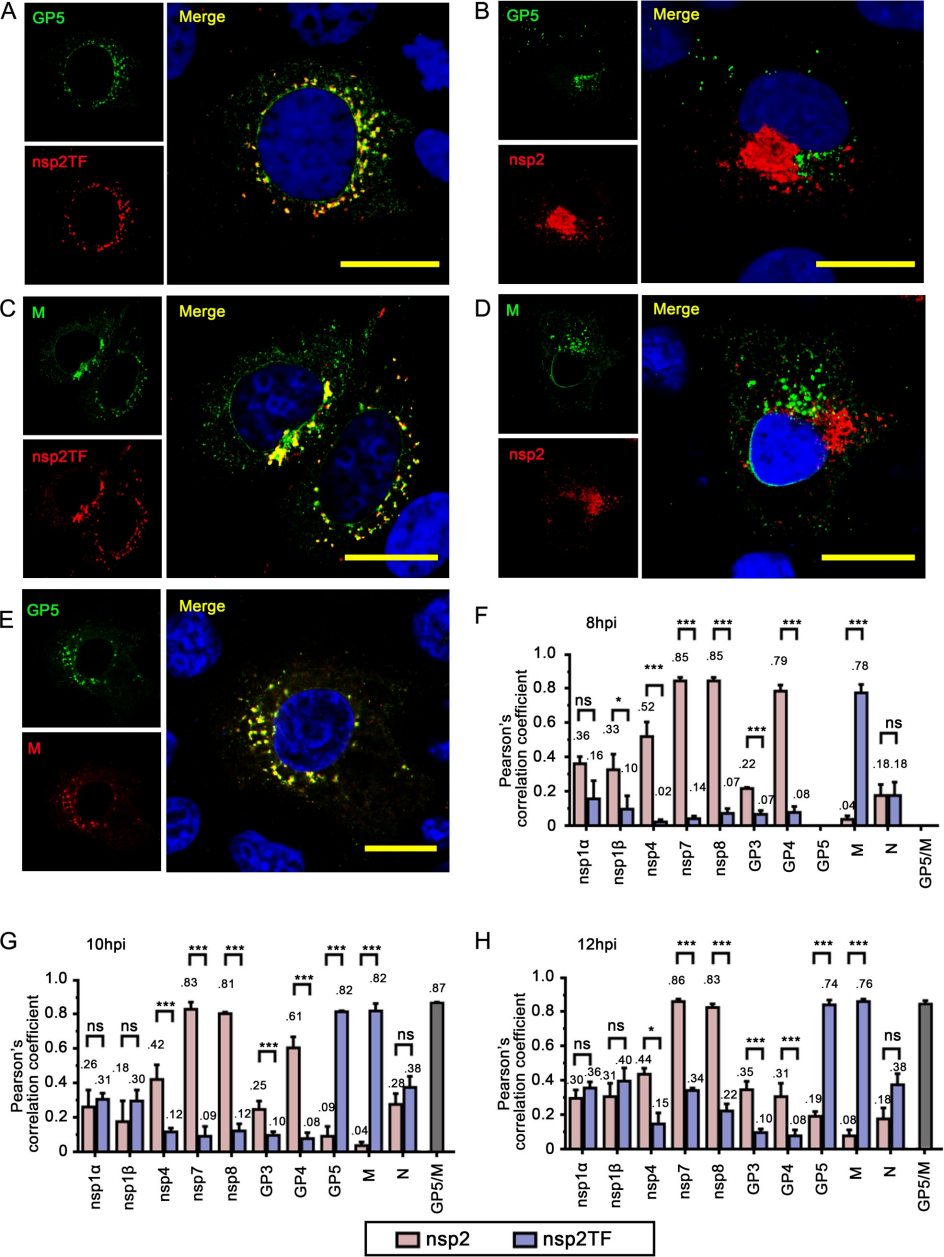
Co-localization analysis of nsp2TF with other PRRSV nonstructural and structural proteins. MARC-145 cells were infected with PRRSV SD95-21 at an moi of 0.1. The cells were fixed at 8, 10 or 12 hpi, permeabilized, and immunostained for nsp1α, nsp1β, nsp4, nsp7, nsp8, GP3, GP4, GP5, M and N proteins together with nsp2TF or nsp2. (**A and B**) Double labeling for GP5 (green) and nsp2TF (**A**) or nsp2 (**B**) in cells fixed at 10 hpi. (**C and D**) Double labeling for M protein (green) and nsp2TF (**C**) or nsp2 (**D**) in cells fixed at 10 hpi. (**E**) Double labeling for GP5 (green) and M protein (red) in cells fixed at 10 hpi. (**F-H**) Analysis of co-localization between nsp1α, nsp1β, nsp4, nsp7, nsp8, GP3, GP4, GP5, M or N protein and nsp2TF or nsp2 in PRRSV-infected MARC-145 cells fixed at 8 (**F**), 10 (**G**), or 12 (**H**) hpi. Nsp2TF or nsp2 foci were chosen as regions of interest for Pearson’s correlation coefficient analysis; 10 foci were randomly selected for each sample (for details, see Materials and Methods). Scale bars, 10μm. ***p < 0.001, *p<0.05. ns, statistically not significant.

To investigate the biological functions of nsp2TF, we first compared its subcellular localization with that of other PRRSV structural and non-structural proteins, including full-length nsp2. Given that the nsp2 and nsp2TF sequences are largely identical, we used polyclonal antibodies that specifically recognize the unique C-terminal segments of nsp2 and nsp2TF. Since the subcellular localization of viral proteins may change over time, we performed a time course experiment. Nsp2TF was first detected at 8 hours post-infection (hpi), showing a strong co-localization with the M protein (Fig 1C). GP5 was not yet detectable at 8 hpi but was clearly detected by 10 hpi (Fig 1A, 1B and 1E). At these early time points, nsp2TF co-localized strongly with both GP5 and M protein (Fig 1A and 1C; Pearson’s correlation coefficient (PCC) > = 0.78, Fig 1F and 1G), but did not co-localize with nsp1α, nsp1β, nsp4, nsp7, nsp8, GP3, GP4, or N protein (PCC < 0.4, Fig 1F and 1G). We could not directly compare the localization of nsp2TF with that of nsp2, as the available specific reagents were both rabbit polyclonal antisera recognizing the C-terminal peptide of the respective protein. However, by comparing the GP5 and M protein labeling patterns in specimens that were also labeled for nsp2 or nsp2TF (Fig 1A–1D), we could conclude that nsp2 and nsp2TF are almost fully separated in the infected cell. At 10 hpi (Fig 1G), the PCC value of co-localization between nsp2TF and GP5 was 0.82, which is much higher than that for nsp2 and GP5 (PCC = 0.09). Similarly, the PCC value for nsp2TF versus M protein was 0.82, whereas the nsp2-M value was only 0.04.

In arterivirus-infected cells, GP5 and M protein form a disulfide-linked heterodimer that is critical for protein transport and progeny virion assembly and egress [1,4,7]. At 10 hpi, the PCC value of GP5 and M protein was 0.87, confirming the co-localization of these two major structural proteins (Fig 1E and 1G). We observed only weak correlations between the localization of nsp2TF and other structural proteins, such as GP3, GP4, or N protein (PCC < 0.4, Fig 1G). Furthermore, the localization of nsp2TF correlated only weakly with those of other nsps tested (PCC < 0.4, Fig 1G). We also analyzed the localization of nsp2TF/nsp2 and GP5/M protein at 12 hpi, which confirmed that nsp2TF continued to co-localize with both GP5 and M protein (PCC > 0.7, Fig 1H). These results demonstrate that nsp2TF and nsp2 have different subcellular localizations, and that most of the nsp2TF, GP5, and M proteins reside in the same membrane compartment(s) of the infected cell at 10–12 hpi.

### Nsp2TF interacts with GP5 and M protein

**Fig 2:**
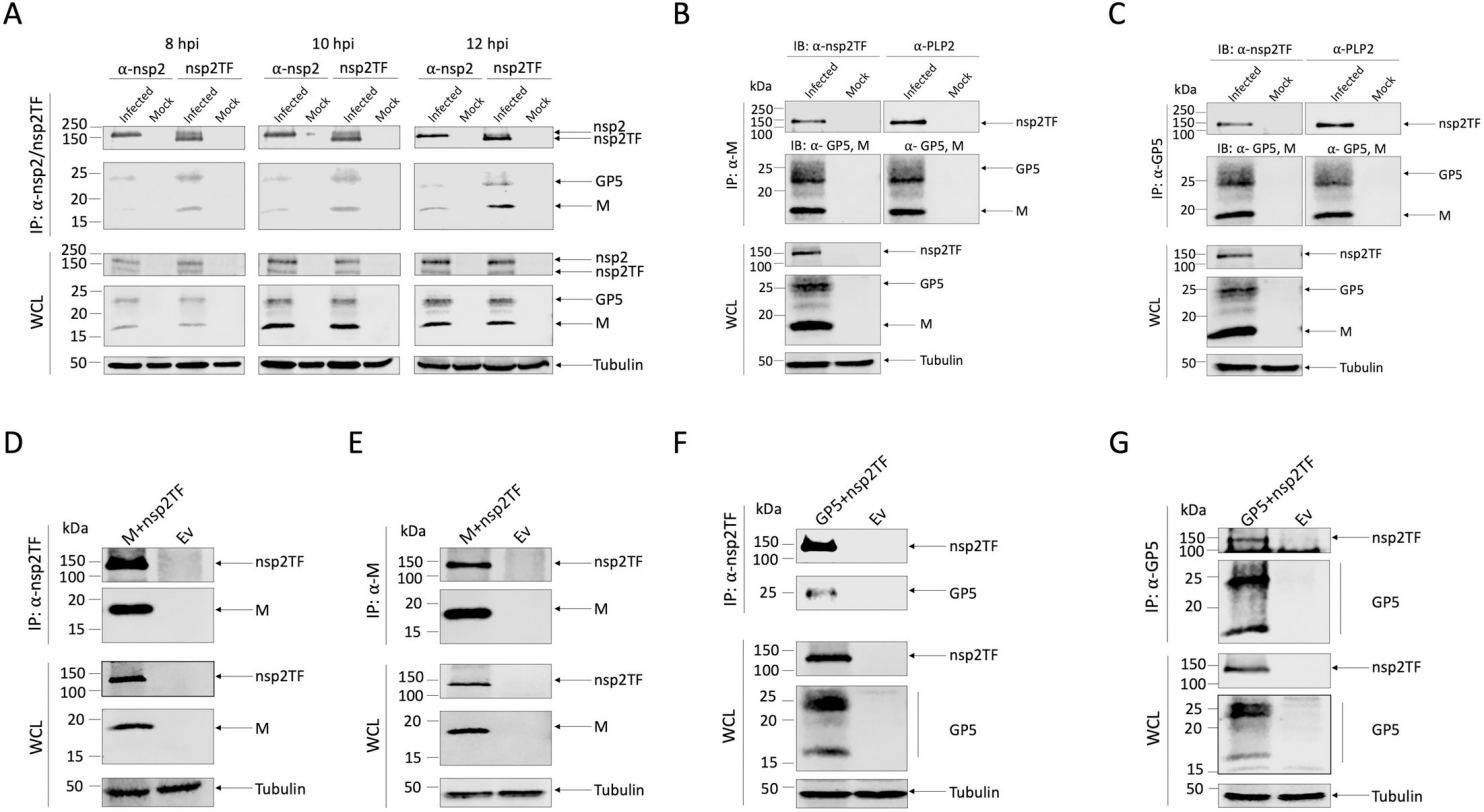
Interaction of nsp2TF with GP5 and M protein. (**A-C**) MARC-145 cells were mock-infected or infected with PRRSV. (**A**) Cell lysates were collected at 8, 10 and 12 hpi and subjected to IP. Lysates were immunoprecipitated with anti-nsp2TF pAb or anti-nsp2 pAb. Western blot analysis was conducted with the whole cell lysate or immunoprecipitated proteins. Membranes were probed with anti-nsp2TF and anti-nsp2 pAbs, or anti-M and anti-GP5 mAbs. (**B-C**) Cell lysates were collected at 12 hpi and subjected to co-IP and western blot analysis. Lysates were immunoprecipitated with anti-M pAb (**B**) or anti-GP5 pAb (**C**) and membranes were probed with anti-nsp2TF pAb, anti-PLP2 mAb, or anti-M and anti-GP5 mAbs. (**D-G**) HEK-293T cells were co-transfected with a plasmid expressing nsp2TF and a plasmid expressing M protein (**D-E**) or GP5 (**F-G**). Cell lysates were collected at 48 h post-transfection and subjected to co-IP using a specific pAb against each individual protein as indicated in each panel. Membranes were probed with anti-nsp2TF pAb, anti-M or anti-GP5 mAb. Anti-tubulin mAb was used as a control. IP: immunoprecipitation; IB: immunoblotting; WCL: Whole cell lysates; Ev: empty vector.

As nsp2TF strongly co-localized with GP5 and M protein, we used co-immunoprecipitation (co-IP) studies to investigate whether nsp2TF interacts directly with the two major viral envelope proteins (or the GP5-M heterodimer). The nsp2 protein was included as a comparison. Lysates from mock-infected or PRRSV-infected MARC-145 cells were collected at 8, 10 and 12 hpi. Half of the sample was used for IP with a rabbit antiserum specific for the TF ORF-encoded C-terminal part of nsp2TF, whereas the other half was used for IP with a rabbit antiserum specific for the C-terminal peptide of full-length nsp2. Immunoprecipitated proteins were probed with antibodies recognizing GP5 or M protein. As shown in Fig 2A and consistent with previous studies [9,20], nsp2 and nsp2TF were detected as protein bands migrating close to the 150 kDa molecular weight marker, with nsp2TF appearing a slightly smaller size than nsp2. The M protein was detected as a protein band migrating close to 20 kDa (the predicted size of M is 18.9 kDa), while GP5 was detected as a collection of bands around 25 kDa, likely due to its heterogeneous glycosylation (the predicted size of non-glycosylated GP5 is 22.4 kDa and each glycan adds ~2.5 kDa to the protein). At 8, 10 and 12 hpi, similar amounts of nsp2 and nsp2TF were detected (the ratios of band intensities of nsp2 relative to nsp2TF were 1.05, 1.11 and 1.04, respectively); however, throughout the time course experiment, nsp2TF pulled down larger amounts of GP5 and M proteins than did nsp2. Based on immunoblot densitometry, the nsp2TF IP brought down 3.39-, 3.26- and 3.36-fold more GP5 than did the nsp2 IP at 8, 10 and 12 hpi, respectively. Similarly, the amount of M protein brought down by the nsp2TF IP was 3.38-, 3.44- and 2.77-fold larger than that in the nsp2 co-IP. This result was consistent with the confocal microscopy analysis (Fig 1) and demonstrated that nsp2TF was strongly associated with the GP5 and M proteins, while a relatively weak association of nsp2 with these envelope proteins was observed.

We subsequently focused on a more detailed analysis of nsp2TF and GP5/M protein interactions. First, we confirmed these interactions by reversing the immunoprecipitation, *i*.*e*. by using an M- or GP5-specific polyclonal antibody (pAb) for the IP and the nsp2TF-specific pAb to probe the Western blot (Fig 2B and 2C). In both cases, clear evidence for co-IP of nsp2TF was obtained, while–as expected–the anti-M antibody also brought down GP5 and vice versa. We further probed the western blot with the monoclonal antibody (mAb) specific for the common PLP2 domain of both nsp2 and nsp2TF. Only nsp2TF was detected in co-IP with M protein (Fig 2B, right panel) or GP5 (Fig 2C, right panel). These data further confirm the strong association of nsp2TF with the GP5 and M proteins. The relatively weak association of nsp2 with these envelope proteins observed in Fig 2A might arise upon cell lysis during the co-IP experiment or from other unknown mechanisms that require future investigation.

Next, we confirmed the observed interactions using ectopic expression of the three interaction partners. Initially, we repeated the co-IP analysis in cells co-transfected with plasmids expressing nsp2TF and GP5 or M protein. In this setup, nsp2TF consistently co-immunoprecipitated with both GP5 and M protein (Fig 2D–2G). We noticed that the anti-GP5 antibody recognized two additional smaller bands (between 15- and 20-kDa) in lysates from cells expressing GP5 (Fig 2F and 2G). These bands likely corresponded to non-glycosylated GP5 species from which the signal peptide has been cleaved, as reported previously [21]. Taken together, these data demonstrated that nsp2TF is able to interact independently with both envelope proteins in their monomeric form.

### Nsp2TF co-localizes with GP5 and M protein along the secretory pathway

**Fig 3:**
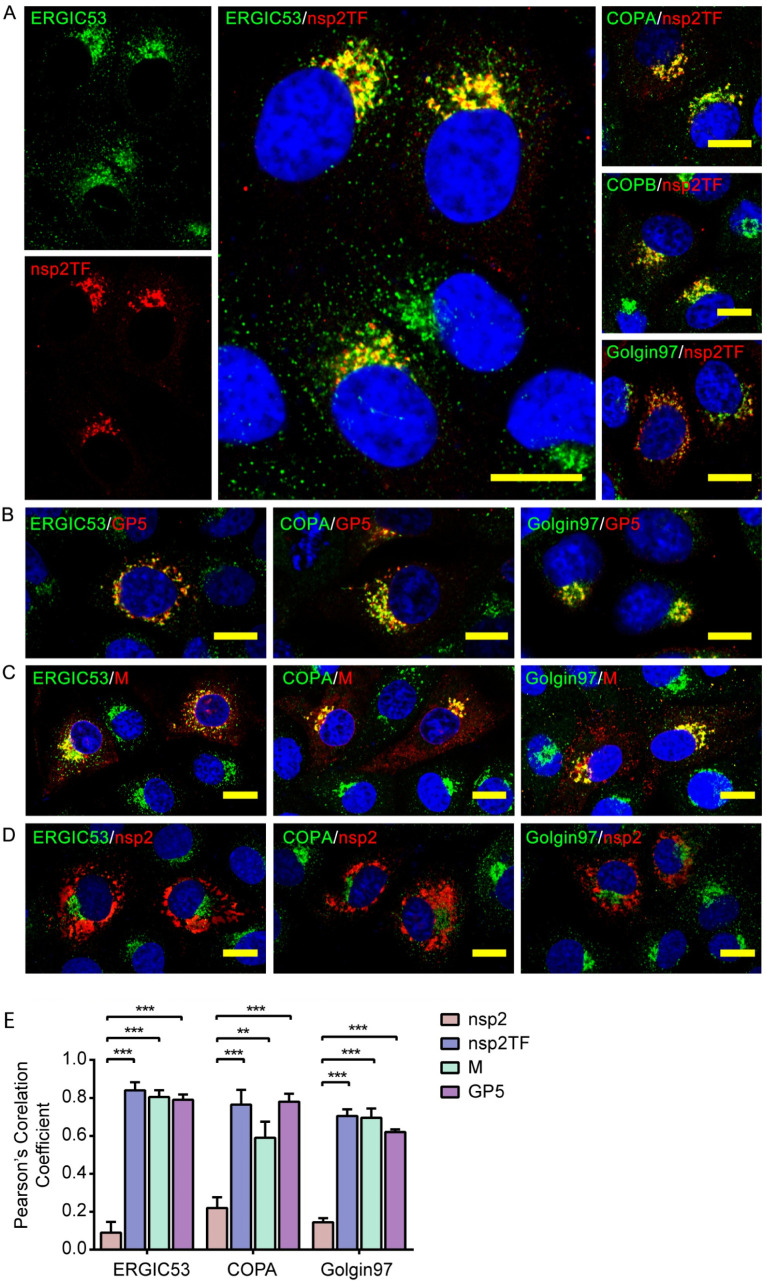
Co-localization analysis between nsp2TF and organelle-specific cellular marker proteins in the secretory pathway. MARC-145 cells were infected with PRRSV SD95-21. At 10 hpi, cells were fixed, permeabilized and immunostained for nsp2TF (**A**), GP5 (**B**), M protein (**C**) and nsp2 (**D**). To label specific cellular compartments, the cells were co-stained with antibodies specific for ERGIC marker proteins ERGIC-53, COPA, COPB, or Golgin 97. Scale bars, 10 μm. (**E**) The level of co-localization between ERGIC makers and nsp2, nsp2TF, GP5 or M protein quantified by Pearson’s correlation coefficient using the Coloc2 module of the ImageJ software package. Mean (± SD) values from three replicates are shown. ***p < 0.001.

Using confocal microscopy and antibodies that recognize organelle-specific marker proteins, we investigated the subcellular (co)localization of nsp2TF, GP5, and M protein in more detail. MARC-145 cells were infected with PRRSV, fixed at 10 hpi, and labeled with antibodies that specifically recognize full-length nsp2, nsp2TF, GP5 or M protein. To visualize the compartments of the secretory pathway, the cells were double-labeled for markers of the ERGIC (including ERGIC-53, COPA, and COPB) and *cis* Golgi (Golgin-97). The results revealed that a major part of the nsp2TF signal co-localized with all of these markers (Fig 3A), indicating that nsp2TF distributes across multiple compartments of the secretory pathway. Both GP5 and M protein also mostly co-localized with ERGIC-53, COPA, and Golgin-97 (Fig 3B and 3C). In contrast, we observed no co-localization between nsp2 and any of the host organelle-specific markers tested (Fig 3D). In line with these observations, the labeling for GP5, M protein, and nsp2TF, but not nsp2, yielded high Pearson’s correlation coefficient values (PCC > 0.5), indicating that these proteins mostly co-localized in defined secretory pathway compartments and/or vesicles (Fig 3E).

### Nsp2TF antagonizes polyubiquitination of GP5 and M protein

Our previous studies revealed that PRRSV mutants lacking nsp2TF expression are viable but crippled, displaying a small-plaque phenotype and 1- to 2-log reduced infectious progeny titers [9,20]. Since we found that nsp2TF co-localizes and interacts with two major players in arterivirus assembly along the secretory pathway, we hypothesized that the protein might somehow promote GP5-M stability or function. As outlined in the introduction, the N-terminal PLP2 domain that is present in both nsp2 and nsp2TF is an established deubiquitinase [14–17], and we recently showed that nsp2TF expression can reverse Ub conjugation from host cell substrates [20]. A recent analysis of the ubiquitome of PRRSV-infected cells established that ubiquitination occurs at several Lys residues of the GP5 and M protein, mostly located in the C-terminal portions of these proteins [22]. We therefore hypothesized that the PLP2 domain of nsp2TF targets the GP5 and/or M protein to reverse their polyubiquitination and thus antagonize their proteasomal degradation.

**Fig 4:**
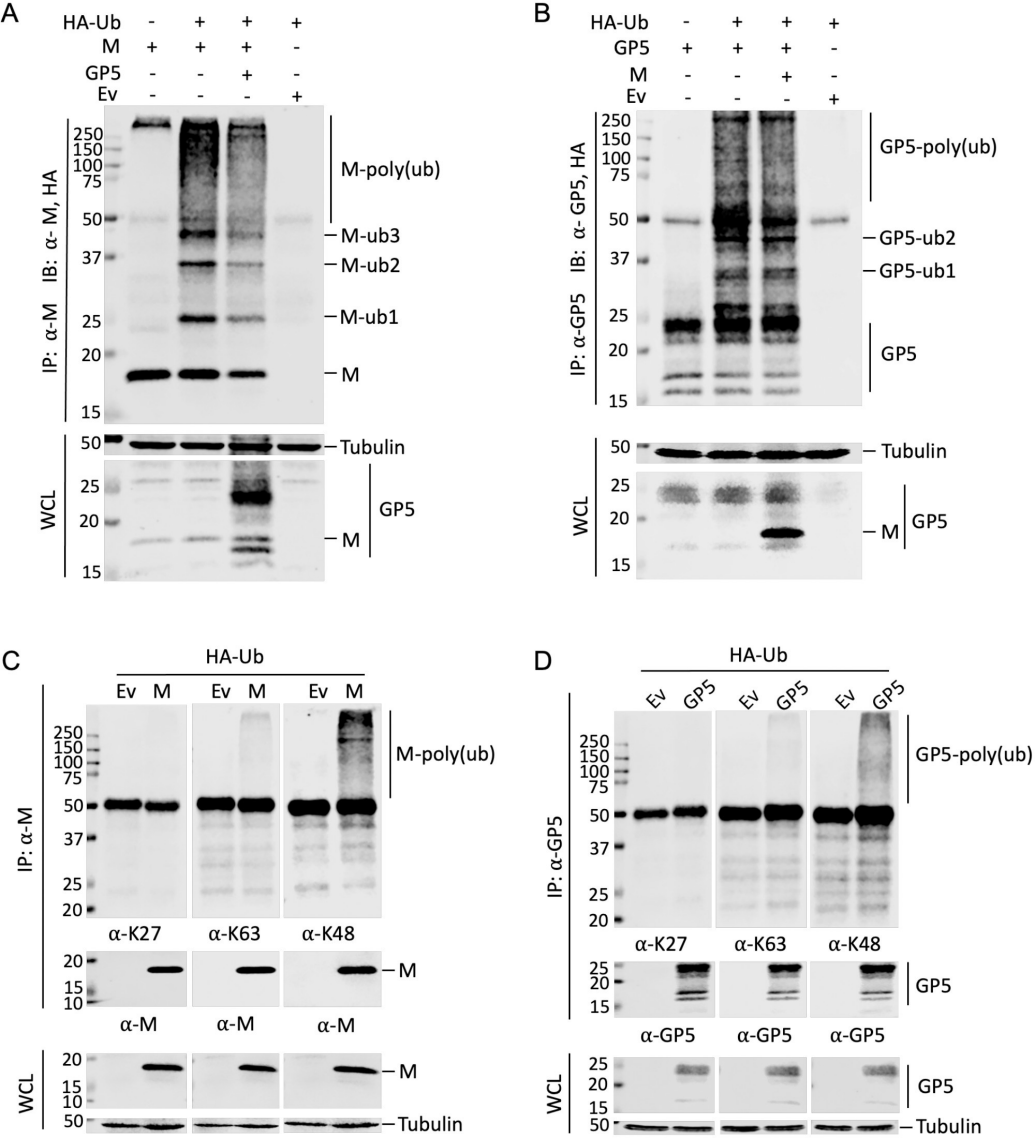
Polyubiquitination of PRRSV GP5 and M protein. HEK-293T cells were co-transfected with plasmids expressing HA-Ub, GP5 and/or M protein. At 42 h post transfection, proteasome inhibitor MG132 was added. At 48 h post transfection, cell lysates were collected for IP analysis. (**A and C**) Lysates were immunoprecipitated with anti-M pAb. (**B and D**) Lysates were immunoprecipitated with anti-GP5 pAb. Western blot analysis was conducted with the whole cell lysate or immunoprecipitated proteins using the mAb against HA, antibodies specifically recognize K27-, K48- or K63-linked polyubiquitin chains, or a specific Ab to each viral protein as labeled for each panel. IP: immunoprecipitation; IB: immunoblotting; WCL: whole cell lysates; Ev: empty vector.

We first determined whether Ub can indeed be conjugated to the GP5 and M protein. HEK-293T cells were co-transfected with plasmids expressing GP5 or M protein, along with HA-tagged Ub or an empty vector (Ev) control. After treatment with the proteasomal inhibitor MG132, immunoprecipitation was performed using a GP5- or M protein-specific antibody, followed by SDS-PAGE and Western blot analysis of Ub conjugates using an anti-HA mAb. In M protein-expressing cells, both in the absence and presence of GP5, we detected a range of Ub-conjugated M protein species, including distinct bands likely corresponding to M proteins (18.9 kDa) modified with one, two, or three Ub copies of ~7 kDa each (Fig 4A). In addition, we detected a number of smeared higher molecular weight bands (Fig 4A), which likely correspond to poly-ubiquitinated M protein species. Although more difficult to interpret directly using Western blot analysis, mainly due to heterogeneous GP5 glycosylation, a similar result was obtained upon expression of GP5. We observed distinct bands with sizes matching the predicted mass of glycosylated GP5 molecules modified with one, two or more Ub moieties (Fig 4B). Again, similar to what was shown in Fig 2, smaller bands between 15- and 20-kDa were consistently observed in cells expressing GP5 [21].

Next, we determined the specific form of ubiquitination in GP5 and M proteins by Western blot using antibodies specifically recognizing K27-, K48- and K63-linked polyubiquitin chain. With the anti-K48-linked polyubiquitin pAb, a prominent smear of ubiquitin-conjugated M (Fig 4C) or GP5 (Fig 4D) proteins were detected. In contrast, only trace amounts of ubiquitin-conjugated M or GP5 protein were detected with the anti-K63-linkage antibody, and no ubiquitin-conjugated M or GP5 protein was detected using the anti-K27-linkage antibody (Fig 4C and 4D). These results indicate that both M and GP5 proteins are primarily modified by K48-linked polyubiquitin.

**Fig 5:**
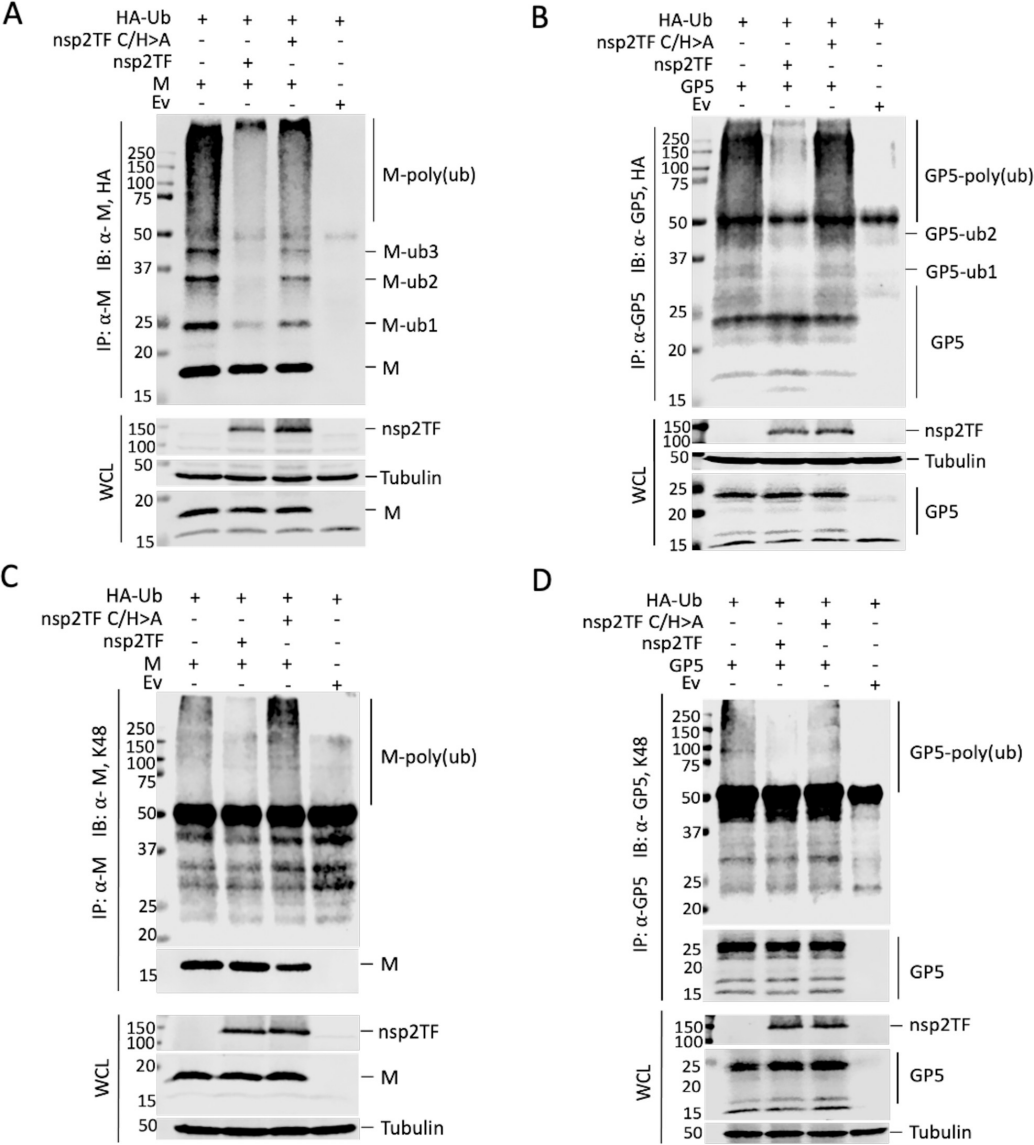
Nsp2TF antagonizes polyubiquitination of GP5 and M protein. HEK-293T cells were co-transfected with plasmids expressing HA-Ub, M protein (**A and C**) or GP5 (**B and D**) and a plasmid expressing nsp2TF or the PLP2 catalytic sites mutant (nsp2TF-C/H>A). Cell lysates were collected at 48 h post transfection. IP was performed with anti-M pAb (**A and C**) or anti-GP5 pAb (**B and D**) and the membrane was probed with anti-HA mAb (**A and B**), a pAb recognizing anti-K48-linked polyubiquitin (**C and D**), or a viral protein-specific antibody. IP: immunoprecipitation; IB: immunoblotting; WCL: whole cell lysates; Ev: empty vector.

Since the GP5 and M protein interact with nsp2TF, we further investigated the impact of DUB activity of the PLP2 domain on their ubiquitination status. HEK-293T cells were co-transfected with a plasmid expressing M or GP5 protein along with a plasmid expressing wild type (WT) nsp2TF or the nsp2TF-C/H>A mutant, in which both catalytic residues (Cys and His) of PLP2 were substituted with Ala to eliminate the DUB activity [20]. Cells that over-expressed nsp2TF-C/H>A mutant showed strongly increased levels of ubiquitinated M and GP5 proteins, while only trace amounts of these species were detected in cells that expressed the WT nsp2TF/PLP2 (Fig 5A and 5B). Use of the pAb specific for K48-linked polyubiquitin revealed that at least part of this increase was due to the accumulation of proteins carrying this extension (Fig 5C and 5D). This result strongly suggested that the PLP2 domain of nsp2TF can directly antagonize the ubiquitination of the PRRSV M and GP5 proteins. This activity is lost upon specific PLP2 inactivation, indicating that PLP2’s DUB capability is critical for this function.

**Fig 6:**
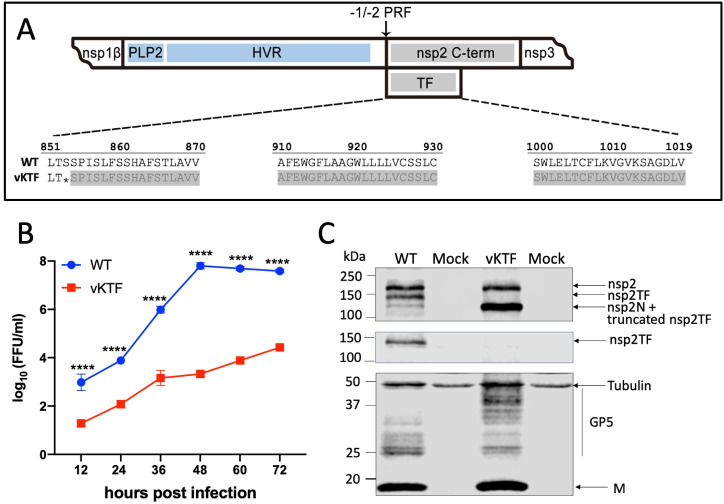
Construction and characterization of PRRSV mutant vKTF. (**A**) Schematic diagram of the engineered nsp2TF truncation. The stop codon introduced in the construct is labeled with an asterisk. The truncated region is shaded with gray. HVR, hypervariable region; nsp2 C-term: nsp2 C-terminal domain that is distinct from that of nsp2TF. (**B-C**) MARC-145 cells were infected with WT virus or its mutant vKTF. Cell lysates were harvested every 12 hpi. (**B**) Growth kinetics comparison of WT virus and vKTF mutant. Each data point represents the mean of three replicates. Virus titers are presented as numbers of fluorescent-focus units (FFU) per milliliter (ml). ****p < 0.0001. (**C**) Cell lysate harvested at 36 hpi was subjected to Western blot analysis using anti-PLP2 mAb that recognizes the common N-terminal PLP2 domain present in all three nsp2 variants (top panel), the TF-specific pAb (middle panel), or mAbs specific for M and GP5 proteins (bottom panel). Anti-tubulin mAb was used as a control.

We further explored the connection between nsp2TF and GP5/M protein ubiquitination in the context of PRRSV-infected cells. In this setting, PLP2 inactivation is not an option, as the nsp2/3 cleavage by PLP2 is critical for virus viability. Instead, we employed a previously described strategy to truncate the TF ORF without affecting ORF1a (which encodes the full-length nsp2), thus creating an nsp2TF-deficient but replication-competent PRRSV mutant [9,10]. This vKTF mutant was constructed by introducing a stop codon that retained only the first two TF ORF-encoded residues (Fig 6A). Using reverse genetics, vKTF mutant virus was recovered from a full-length cDNA infectious clone of PRRSV-2 strain SD 95–21. Growth kinetics analysis showed that this virus mutant had about 3-log reduced peak viral titer compared to wild type (WT) virus (Fig 6B). Western blotting detected the expression of GP5 and M protein in both WT virus and vKTF mutant infected cells (Fig 6C). The result confirmed that full-length nsp2TF was not expressed in vKTF-infected cells. The truncated nsp2TF is only two amino acids longer than nsp2N (Fig 6A), whose function in infected cells has remained elusive thus far. Accordingly, we detected a substantially larger amount of proteins at the size of nsp2N in vKTF-infected cells compared to the WT virus (Fig 6C).

**Fig 7:**
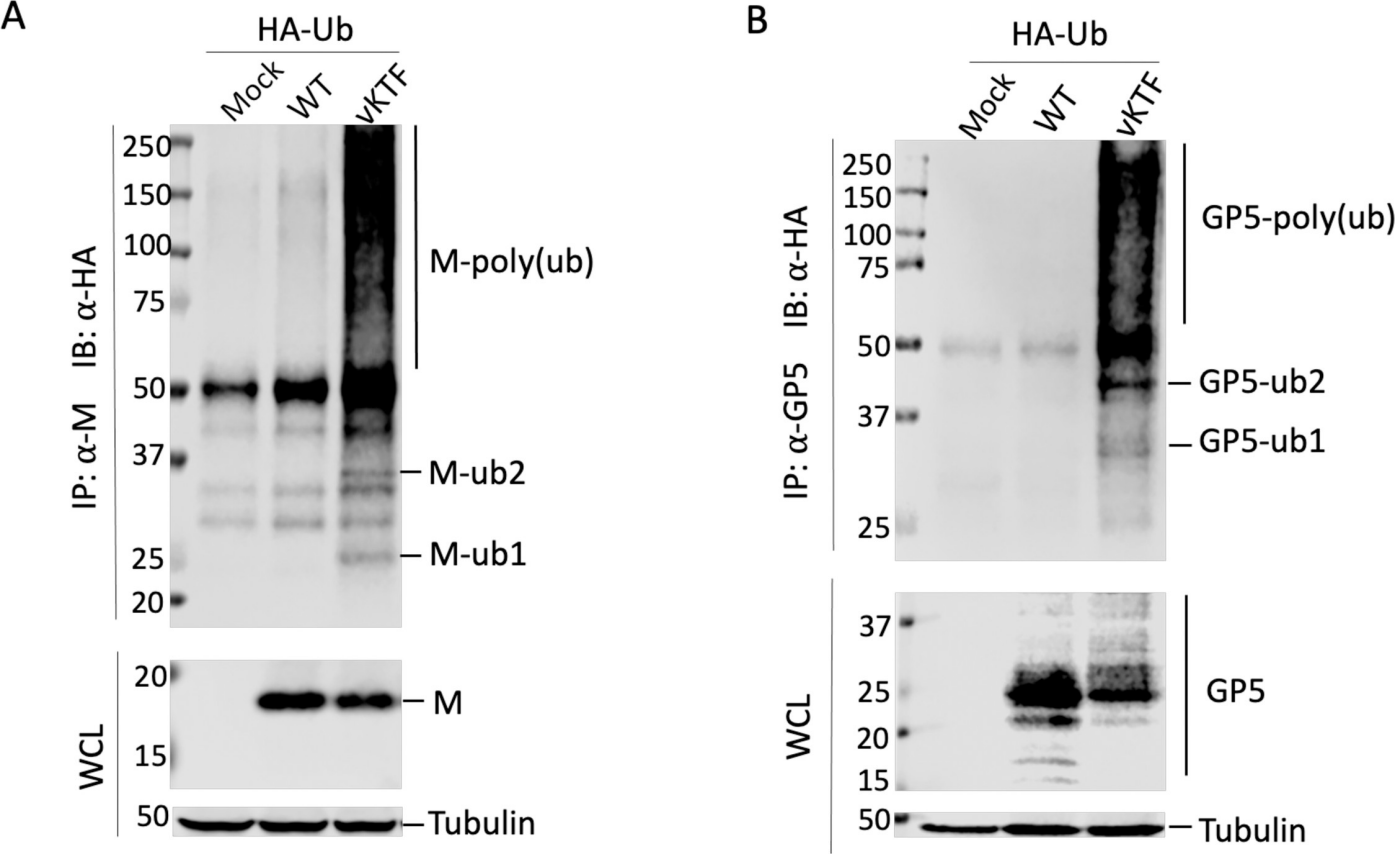
Nsp2TF deubiquitinating GP5 and M protein in the context of PRRSV-infected cells. (**A-B**) HEK-293T cells were co-transfected with a plasmid expressing HA-Ub and a PRRSV full-length infectious cDNA clone of WT virus or its mutant vKTF. The cell lysates were collected at 48 h post-transfection. IP was performed with anti-M pAb (**A**) or anti-GP5 pAb (**B**). Immunoprecipitated proteins were separated by SDS-PAGE and analyzed by Western blot using anti-HA mAb (**A-B**). Western blot analysis was also conducted with pre-IP whole cell lysates using M-specific mAb (**A**) or GP5 specific mAb (**B**). Tubulin was detected as a loading control. IP: immunoprecipitation; IB: immunoblotting; WCL: whole cell lysates.

Next, we determined the ubiquitination status of M and GP5 proteins in WT virus or vKTF mutant infected cells. Initially, we tried to detect endogenous levels of M and GP5 ubiquitination in PRRSV-infected MARC-145 cells or swine macrophages. However, we were unable to find a commercial antibody that reliably recognizes endogenous Ub in these PRRSV permissive cells. As an alternative approach, the ubiquitination status of M and GP5 proteins was analyzed in HEK-293T cells that were co-transfected with a plasmid expressing HA-Ub and a full-length PRRSV cDNA infectious clone from which WT or vKTF mutant virus can be launched. The expression and ubiquitination status of M and GP5 proteins were evaluated by immunoprecipitation and Western blot assay using specific antibodies as indicated in Fig 7A and 7B. As expected, in cells transfected with the WT PRRSV cDNA infectious clone, M and GP5 proteins were detected at their predicted sizes and ubiquitinated M and GP5 proteins were almost undetectable. In contrast, much larger amounts of ubiquitinated M and GP5 proteins (mono-, di-, and poly-ubiquitinated) were present in cells transfected with the vKTF mutant expressing the truncated form of nsp2TF. In line with the higher levels of ubiquitinated viral proteins, the levels of unmodified M and GP5 proteins were clearly decreased, demonstrating a shift to ubiquitinated forms (Fig 7A and 7B). Taken together, these data indicate that M and GP5 proteins are abundantly ubiquitinated in both an ectopic expression system and virus-infected cells, and that their Ub conjugation can be reversed by nsp2TF through its PLP2 DUB activity.

**Fig 8:**
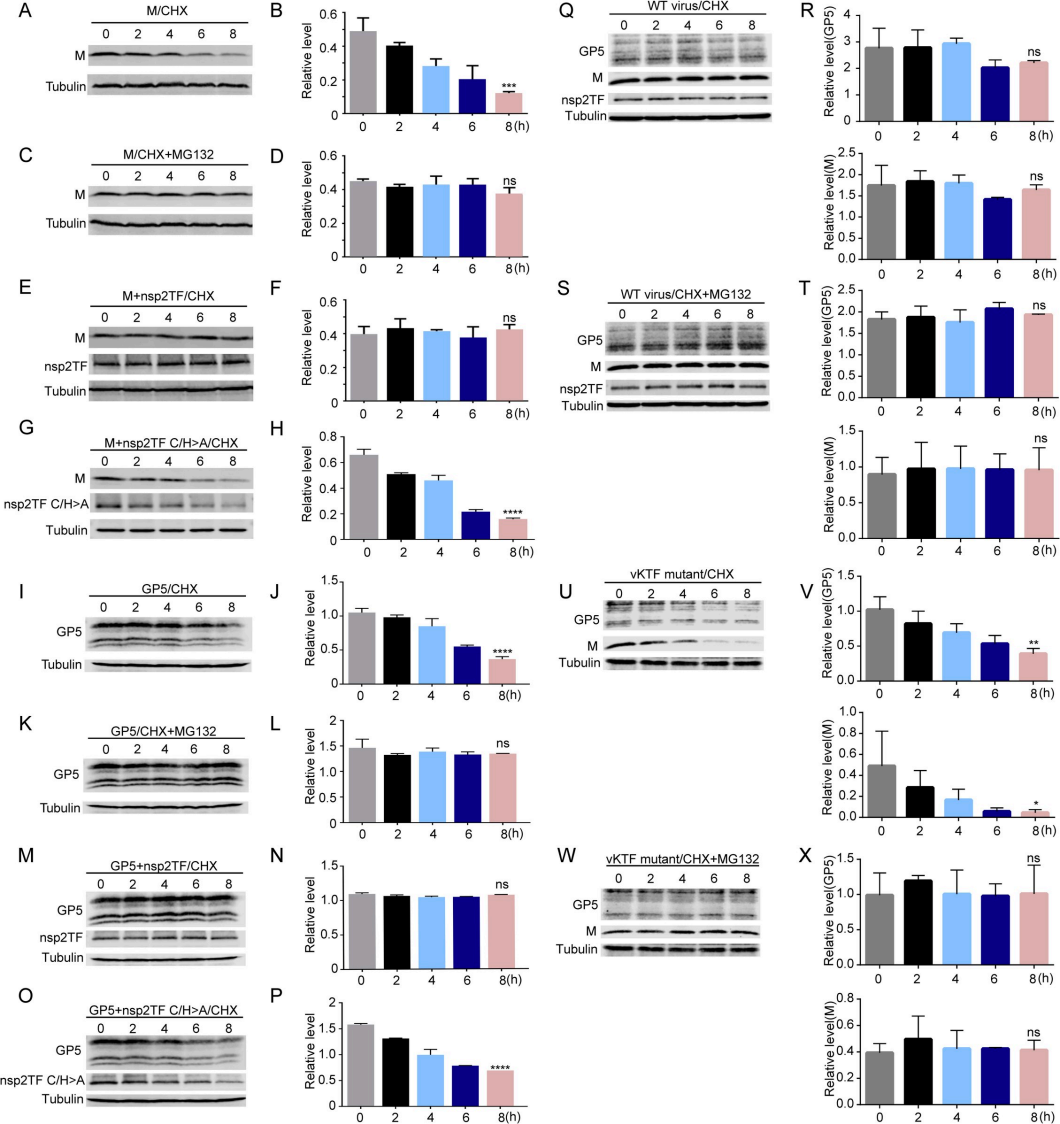
Nsp2TF protects GP5 and M protein from UPP degradation. (**A-P**) HEK-293T cells were transfected with a plasmid expressing GP5 or M protein, or co-transfected with plasmids expressing the nsp2TF (or its mutant nsp2TF C/H>A) and GP5 or M protein. At 24 h post-transfection, transfected cells were treated with CHX alone or in combination with the proteasome inhibitor MG132. For each transfection condition, cells were collected every 2 h after the drug treatment. The whole cell lysates were used for Western blot analysis. Nsp2TF, GP5 and M protein expression were detected with anti-nsp2TF pAb, anti-M pAb and anti-GP5 mAb. β-tubulin was included as a loading control. (**B, D, F, H)** Relative protein expression levels of M protein under the corresponding experimental condition in the left panels A, C, E, G, respectively. **(J, L, N, P**) Relative protein expression levels of GP5 under the corresponding experimental condition in the left panels I, K, M, O, respectively. (**Q, S, U, W**) MARC-145 cells were infected with WT virus (panels Q and S) or mutant vKTF (panels U and W). At 12 hpi, the cells were treated with CHX alone or together with MG132. Cell lysates were collected every 2 h. The whole cell lysates were used for Western blot analysis. Viral protein expression was detected with a specific antibody as described above. (**R, T, V, X**) The relative expression levels of GP5 and M protein under the experimental conditions in panels Q, S, U, W, respectively. Each graph for relative protein expression level was built based on two independent experiments. ****p < 0.0001, ***p < 0.001, **p < 0.01, *p < 0.05. ns, statistically not significant.

Since both GP5 and M proteins are primarily modified with K48-linked polyubiquitin that is typically associated with proteasome-mediated degradation, we confirmed whether M and GP5 protein levels are regulated through the ubiquitin-proteasome pathway (UPP). HEK-293T cells were single-transfected with a plasmid expressing the M or GP5 protein or double-transfected with plasmids expressing M/GP5 and nsp2TF. At 24 h post-transfection, cells were treated with either cycloheximide (CHX) alone to block protein synthesis or both CHX and MG132 to block both protein synthesis and proteasomal activity. The protein turnover dynamics of M protein or GP5 were monitored for up to 8 hours. In cells not expressing nsp2TF, M protein or GP5 was found to be steadily degraded. In the 8 h following the start of CHX treatment, M protein levels decreased 4.45-fold (Fig 8A and 8B). GP5 showed three major bands as described above, including the ~25 kDa glycosylated protein and two smaller-size products likely representing species from which the signal peptide has been removed. Compared to that at the start point of CHX treatment, the total protein level of these three GP5 species decreased 2.87-fold in 8 h (Fig 8I and 8J). The addition of MG132 stabilized the levels of M protein (Fig 8C and 8D) and GP5 (Fig 8K and 8L), indicating that these viral proteins were otherwise degraded via the UPP. Importantly, when nsp2TF was co-expressed, M and GP5 protein levels remained constant throughout the time course of this experiment, even in the absence of proteasomal inhibition (Fig 8E, 8F, 8M and 8N). To further confirm the role of PLP2 in this result, we co-expressed the nsp2TF-C/H>A mutant containing an inactivated PLP2 DUB. In this set-up, the M and GP5 proteins were found to be steadily degraded with 4.16-fold and 2.27-fold decrease of protein levels in 8 h, respectively (Fig 8G, 8H, 8O and 8P), which is similar as in cells expressing the M protein or GP5 alone (Fig 8A, 8B, 8I and 8J).

To confirm the result obtained with the ectopic expression system, we further investigated GP5/M protein degradation in MARC-145 cells infected with vKTF mutant or WT virus. At 24 hpi, cells were treated with CHX alone or with a combination of CHX and MG132. In the WT virus-infected cells, little or no degradation of M or GP5 protein was observed after CHX treatment through the time course of the study, indicating that the M and GP5 proteins are protected from rapid UPP degradation (Fig 8Q–8T). In vKTF mutant-infected cells, however, M and GP5 protein levels decreased gradually following the start of CHX treatment, to approximately 10.51-fold (M) or 2.54-fold (GP5) lower level after 8 hours (Fig 8U and 8V). As in the expression system, MG132 treatment stabilized the level of M and GP5 proteins (Fig 8W and 8X). Our combined data thus strongly support the hypothesis that nsp2TF can interfere with the UPP-driven turnover of the PRRSV M and GP5 proteins by virtue of the DUB activity of its PLP2 domain.

## Discussion

PRRSV nsp2 is a multi-functional replicase subunit, of which different domains play different roles in viral replication and pathogenesis [8,14,15,20,23]. The discovery of PRF-driven nsp2TF and nsp2N synthesis has added to the complexity of the functions encoded in this region of the viral genome [9]. Full-length nsp2, nsp2TF, and nsp2N all contain an identical N-terminal segment that includes the PLP2 domain and the so-called ‘hypervariable region’ [1,8,9]. Nsp2N is thought to be cytosolic, and technically difficult to study due to its exact collinearity with both nsp2 and nsp2TF. In contrast, nsp2 and nsp2TF are transmembrane proteins with different C-terminal hydrophobic domains. Our microscopic studies demonstrated that nsp2 and nsp2TF localize to different subcellular compartments of PRRSV-infected cells, a difference that is likely linked to the properties of their respective transmembrane domains. Whereas the full-length nsp2 resides primarily in the ER-derived (double) membranes of the viral replication organelles [12,13], nsp2TF localizes to compartments of the exocytic pathway. This difference strongly suggests that full-length nsp2 and nsp2TF have different biological functions during PRRSV replication. We found that nsp2TF, but not nsp2, is targeted to the same compartments of the secretory pathway (ERGIC and Golgi), in which the two major envelope proteins of PRRSV, GP5 and M, accumulate. The details of PRRSV assembly, and its exact subcellular localization in particular, have not yet been elucidated. However, the formation of a disulfide-linked GP5-M heterodimer and its interaction with the nucleocapsid structure are generally assumed to be the key events that trigger virion budding from ‘smooth membranes’ along the secretory pathway, as observed by electron microscopy for various arteriviruses [1]. The co-localization and interaction of nsp2TF with GP5/M protein in the secretory pathway and the ability of nsp2TF to inhibit GP5/M protein turnover now indicate an important role for nsp2TF in promoting arterivirus assembly. We propose that the targeting of nsp2TF to the sites of PRRSV assembly stabilizes the protein levels of M, GP5, and/or GP5-M by virtue of the DUB activity of the nsp2TF PLP2 domain. This hypothesis is clearly supported by the previously described phenotype of multiple nsp2TF-deficient PRRSV mutants, which generally display normal RNA and nsp synthesis [10], but a small-plaque phenotype and reduced infectious progeny titers [9,10]. These phenotypic features are consistent with a specific reduction of virus assembly due to increased GP5/M protein turnover caused by a lack of nsp2TF expression.

Thus far, the DUB activity of the arterivirus PLP2 protease has mainly been investigated in the context of viral innate immune evasion. PRRSV PLP2 has been demonstrated to be involved in inhibiting NF-κB activation in infected host cells by interfering with the polyubiquitination of IκBα, which in turn suppresses the host innate immune response [14]. The DUB activity of the EAV PLP2 domain was shown to target critical ubiquitination-dependent factors in the host cell’s innate immune response, although specific substrates remain to be identified [18]. Recent studies demonstrated that the PRRSV PLP2 domain also functions as a DUB when expressed as part of nsp2TF, and is able to antagonize cellular protein ubiquitination and suppress innate immune responses [20]. We now demonstrate that the PLP2-DUB domain of nsp2TF also targets viral substrates, specifically to reduce proteasomal degradation of GP5 and M protein. Consequently, when nsp2TF expression is knocked out (as in mutant vKTF), viral replication is feasible, but the mutant is severely crippled. This indicates that nsp2TF is dispensable for basic virus viability, but important for achieving maximal PRRSV fitness. Our data also suggest that the levels of GP5 and M proteins are rate-limiting factors in the assembly of infectious arterivirus progeny.

The ubiquitin-proteasome pathway (UPP) has essential antiviral functions *in vivo*. Viral proteins synthesized in infected cells are partially degraded through the proteasome. The resulting peptides, bound to major histocompatibility complex (MHC) class I molecules, are transported to the cell surface and presented to the host immune system, which triggers specific antiviral responses [24]. Recently, nsp2TF expression was reported to downregulate the expression of Swine Leukocyte Antigen class I (SLA-I) [25]. Interestingly, MHC class I molecules also travel along the exocytic pathway and may interact, directly or indirectly, with nsp2TF molecules that reside in the same membrane compartments. A mutagenesis study showed that the C-terminal 68 aa of nsp2TF is essential for SLA-I downregulation. Although the localization of this truncated nsp2TF mutant was not studied, the protein may well be mis-targeted or unstable, as it lacks 2 of the 4 predicted transmembrane segments of the TF domain. Likewise, the truncated nsp2TF variant expressed by the PRRSV mutant vKTF used in this study may not achieve the localization or membrane topology of its wild-type counterpart. As the truncation deletes the epitope recognized by our nsp2TF-specific antibody, we were unable to investigate this in more detail in the current study.

To propagate themselves optimally, viruses have evolved multiple strategies to avoid or reverse ubiquitination of their critical proteins, including encoding lysine-free proteins and DUBs in their genomes [19]. Numerous viral DUBs have been described from an array of DNA and RNA viruses, including herpes simplex virus 1, PRRSV and EAV, severe acute respiratory syndrome coronaviruses (SARS-CoV) and other coronaviruses [14–19,26]. These DUBs play essential roles in the suppression of host immune responses by removing Ub conjugates from cellular substrates involved in immune gene activation. Currently, there are very few documented examples of viral DUBs that are used to protect viral proteins from the UPP pathway. Previously, the DUB domain of the 98K replication protein of the plant RNA virus turnip yellow mosaic virus (TYMV) was reported to counteract the UPP-mediated degradation of its own RNA-dependent RNA polymerase (RdRp), which contributes to viral infectivity in plant cells [27]. To our knowledge, this is the first study to demonstrate that a viral DUB can be used to counteract the ubiquitination and degradation of key viral structural proteins in order to promote the production of viral progeny. This novel mechanism may also be utilized by other viral DUBs and warrants further investigation.

Since nsp2TF associates with GP5-M protein in the exocytic pathway, it is possible that nsp2TF may also function as a chaperone or scaffold to facilitate the maturation or heterodimerization of these envelope proteins. Also, in this manner, PRRSV nsp2TF may promote GP5-M protein stability and integrity in the early secretory pathway. Some viruses encode specialized viral chaperones to facilitate their replication and propagation [28,29]. PRRSV nsp2 has been reported to interact with numerous cellular chaperones in the ER [30–32]. Since nsp2TF shares an identical N-terminal segment with nsp2, nsp2TF may also recruit such cellular chaperones in the ER to promote the maturation of structural proteins and, eventually, the assembly of new virions. Moreover, association with nsp2TF may itself prevent GP5-M protein aggregation or destabilization, by directly blocking aggregation or destabilization-prone domains on these proteins.

In summary, the newly identified -2 PRF product, nsp2TF, is a multi-functional protein. It has at least three biological functions: suppressing host innate immune response, influencing MHC-I expression, and stabilizing major viral envelope proteins. A more detailed understanding of these functions will enhance our insight into PRRSV pathogenesis, which will provide the basis for developing more effective control strategies.

## Materials and methods

### Cells and viruses

HEK-293T and MARC-145 cells were maintained in minimum essential medium (MEM, Gibco) supplemented with 10% fetal bovine serum and antibiotics (Penicillin and Streptomycin, 100 μg/ml) at 37 ^o^C with 5% CO_2_. PRRSV-2 isolate SD95-21 (GenBank accession #KC469618) and its full-length cDNA infectious clone, pCMV-SD95-21, were used for all experiments in this study.

A PRRSV mutant that expresses truncated nsp2TF protein was engineered by introducing a stop codon in the TF ORF using nucleotide substitutions that are translationally silent with respect to ORF1a, in order to not affect the sequence of the full-length nsp2. Using our previously described approach [9,10], nsp2TF (1019 aa long in SD95-21) was truncated after residue 852 in mutant vKTF (Fig 6A). The mutant virus was recovered by reverse genetics and viral growth kinetics was compared with WT virus using the methods as we described previously [9,10].

### Antibodies

Table 1 lists the polyclonal and monoclonal antibodies used in this study. Most of the PRRSV-specific antibodies were described previously [9,10,33–37]. Rabbit polyclonal antiserum (pAb anti-M), recognizing the C-terminal peptide (LKSLVLGGRKAVK) of the PRRSV-2 M protein, and rabbit polyclonal antiserum (pAb anti-GP5), recognizing the C-terminal peptide (VATPITRVSAEQWG) of the PRRSV-2 GP5 protein, were generated by Genscript (Piscataway, NJ). Monoclonal antibody (mAb 120–60) against the M protein was generated by immunizing BALB/c mice with the M peptide (LKSLVLGGRKAVK) conjugated to Keyhole Limpet Hemocyanin (KLH). Antibodies obtained from commercial resources are summarized in Table 1.

**Table 1:** List of primary antibodies used in this study.

Source	Antibody name*	Protein target	Predicted molecular mass of target protein (kDa)	References
**PRRSV-2 (SD95-21)**	mAb 73–14	nsp1α	19.9	[36]
	mAb 123–128	nsp1β	23	[37]
	pAb anti-nsp2TF	nsp2TF (epitope CFLKVGVKSAGDLV)	110.1	[9, 10]
	pAb anti-nsp2	nsp2 (C-terminus) (epitope NGLKIRQISKPSGG)	129.4	[35]
	mAb 140–68	PLP2	129.4	[10]
	mAb 69–267	nsp4	21	[35]
	mAb 108–16	nsp7	28.6	[35]
	mAb 101–48	nsp8	4.9	[35]
	mAb 120–29	GP3	29.1	[36]
	mAb NI37	GP4	19.5	[34]
	mAb 21–79	GP5^#^	22.4	[35]
	mAb 120–60	M	18.9	This study
	pAb anti-M	M (C-terminus) (epitope LKSLVLGGRKAVK)	18.9	This study
	pAb anti-GP5	GP5^#^ (epitope VATPITRVSAEQWG)	22.4	This study
	mAb SDOW17	N	13.6	[33]
**Cellular Proteins**	mAb C-6	ERGIC 53	57.5	Santa Cruz
	pAb E1030	ERGIC 53	57.5	Sigma
	pAb PA1-061	COPB	107.1	Thermo Fisher
	mAb H-3	COPA	138.3	Santa Cruz
	mAb D-10	COPB	107.1	Santa Cruz
	mAb CDF4	Golgin 97	88.2	Santa Cruz
	Golgin 97 Antibody (CDFX) Alexa Fluor 594	Golgin 97	88.2	Santa Cruz
	pAb #3393	Ubiquitin	8.5	Cell Signaling Technology
	mAb H-235	β- tubulin	50	Santa Cruz
	mAb EPR17034	K27-linkage-specific Polyubiquitin	8	Abcam
	pAb #4289	K48-linkage-specific Polyubiquitin	8	Cell Signaling Technology
	mAb #5621	K63-linkage-specific Polyubiquitin	8	Cell Signaling Technology
**Tag**	mAb 16B12	HA	3.6	Biolegend

*mAb: monoclonal antibody; pAb: polyclonal antibody.

#Predicted molecular mass of GP5 was calculated based on the amino acid sequence of GP5 without including the size of glycan.

### Plasmids

Plasmids pCAGGS-GP5 and pCAGGS-M, expressing untagged GP5 and M protein, were constructed by cloning nucleotides 13789–14391 and 14376–14900 of the PRRSV-2 SD95-21 genome, respectively, into eukaryotic expression plasmid vector pCAGGS-MCS (a generous gift from Dr. Adolfo Garcia-Sastre at the Icahn School of Medicine at Mount Sinai in New York City). The pCAGGS-nsp2TF plasmid was constructed by PCR amplification of the nsp2TF-coding region as described previously [20]. The PCR product was cloned into vector pCAGGS-MCS. Plasmid pCAGGS-nsp2TF-C/H>A was constructed by introducing Ala substitutions at PLP2 active site residues Cys433 and His503 in pCAGGS-nsp2TF. Plasmid pcDNA3.1-HA-Ub, which was used in ubiquitin (Ub) conjugation assays, was obtained from Addgene (Plasmid #18712; Watertown, MA).

### Immunofluorescence microscopy

Immunofluorescence assays were performed as described previously [35,36]. To detect PRRSV proteins, specific mAbs or pAbs listed in Table 1 were used. To detect cellular marker proteins within distinct compartments of the secretory pathway, infected cells were stained with anti-ERGIC-53 mAb (C-6), anti-COPA mAb (H-3), anti-COPB mAb (D-10), or anti-Golgin-97 mAb (CDFX) (Santa Cruz, Dallas, TX). Anti-ERGIC-53 pAb (E1030, Sigma-Aldrich, St. Louis, MO), anti-COPA pAb (PA1-061, Thermo Fisher Scientific, Waltham, MA), and Alexa-fluor 594 conjugated anti-Golgin 97 mAb (CDFX, Santa Cruz, Dallas, TX) were used along with GP5 mAb for co-localization analysis. After a 1h incubation at 37°C, Alexa Fluor 488 AffiniPure goat anti-mouse IgG (Jackson ImmunoResearch, West Grove, PA) or Alexa Fluor 594 AffiniPure goat anti-rabbit IgG (Jackson ImmunoResearch, West Grove, PA) was added as the secondary antibody. After an additional 1h incubation at room temperature, cells were washed in 1x PBS and then stained with 4’,6-diamidino-2-phenylindole (DAPI) (Invitrogen, Carlsbad, CA). Confocal microscopy was performed using a Zeiss LSM880 confocal laser-scanning microscope. The fluorescence intensities of selected regions were measured using Zen 2 software (Carl Zeiss, Oberkochen, Germany).

Co-localization was quantified by choosing nsp2TF- or nsp2-containing foci as regions of interest (ROI) and calculating Pearson’s correlation coefficient (PCC) with the Coloc 2 plugin of image processing program ImageJ (*https*:*//imagej*.*net/Fiji*). This software implements and performs the pixel intensity correlation over space method of Pearson. The PCC is calculated based on the spatial correlations of the intensity distributions of two different fluorescent signals over all the pixels of a ROI. As the PCC is normalized by the standard deviations of the respective intensity distributions, its value ranges from 1 (perfectly correlated intensities) to zero (randomly / uncorrelated intensities) to -1 (completely anticorrelated intensities). A high PCC (0.5–1) indicates meaningful colocalization of two fluorescent species. To apply Coloc2 for colocalization analysis, 10 ROIs containing nsp2 or nsp2TF positive signal were randomly selected for each cell, and a PCC analysis of the ROIs was performed with Coloc 2 to assess the correlation between other viral proteins and nsp2TF or nsp2.

### Immunoprecipitation (IP) and Western blot analysis

IP was performed using methods described previously [9,35]. To analyze interactions between GP5, M protein and nsp2TF or nsp2, whole lysates of PRRSV-infected cells were harvested and IP was initially performed using an nsp2TF or nsp2-specific pAb that recognizes the unique C-terminal domain of nsp2TF or nsp2. To study interactions in more detail, co-IP was performed using anti-GP5 pAb or anti-M pAb. Mock-infected cell lysates were used as controls. The interactions between GP5, M protein, and nsp2TF were further confirmed using an eukaryotic expression system. HEK-293T cells were co-transfected with plasmid pCAGGS-GP5 and/or pCAGGS-M, along with plasmid pCAGGS-nsp2TF. The empty pCAGGS vector was used as a negative control. At 48 h post-transfection, cell lysates were harvested and used for IP analysis with protein-specific antibodies. The immunoprecipitated proteins were separated by SDS-PAGE using reducing gels (40% ProtoGel (37.5:1 Acrylamide:Bisacrylamide), 10% SDS, 1.5M Tris-HCl, National Diagnostics] and transferred to a nitrocellulose membrane for Western blot analysis.

Western blotting was performed as previously described [38]. To detect viral proteins, membranes were probed with primary antibodies against GP5 (mAb 21–79), M protein (mAb 120–60), nsp2TF (pAb anti-nsp2TF), nsp2 (pAb anti-nsp2), or PLP2 (mAb 140–68). The expression of β-tubulin was monitored as a loading control. IRDye 680-conjugated goat anti-rabbit IgG (H+L) and/or IRDye 800CW-conjugated goat anti-mouse IgG (H+L) (LI-COR Biosciences, Lincoln, NE) were used as secondary antibodies. The blots were imaged using an Odyssey infrared imaging system (LI-COR Biosciences).

### Ubiquitination and de-ubiquitination assays

To analyze the ubiquitination status of the PRRSV GP5 and M proteins, HEK-293T cells were co-transfected with plasmids pcDNA3.1-HA-Ub, pCAGGS-GP5 and/or pCAGGS-M. To evaluate nsp2TF-driven de-ubiquitination, cells were also co-transfected with pCAGGS-nsp2TF or pCAGGS-nsp2TF-C/H>A. At 42 h post-transfection, cells were treated with the proteasome inhibitor MG132 (20 μM, Millipore) for an additional 6 h. At 48 h post-transfection, whole cell lysates were harvested using lysis buffer (2% SDS, 150 mM NaCl, 10 mM Tris-HCl, protease/phosphatase inhibitor cocktail, 5 mM N-ethylmaleimide, pH 8.0). Cells were further disrupted using an ultrasonic homogenizer. After sonication, cell lysate was heated at 96°C for 5 min and then mixed with 9 volumes of dilution buffer (150 mM NaCl, 10 mM Tris-HCl, protease/phosphatase inhibitor cocktail, 5 mM N-ethylmaleimide, pH 8.0). The diluted cell lysate was incubated at 4°C for 30 min with rotation. Cell debris was then removed by centrifugation at 4°C. The cleared supernatant was used for immunoprecipitation with anti-GP5 pAb or anti-M pAb. Ubiquitin-conjugated proteins were separated by SDS-PAGE and blotted with anti-HA mAb or rabbit anti-sera specifically recognizing K27-, K48-, or K63-linked polyubiquitin. Western blot analysis was also performed with specific mAb to detect GP5 or M protein.

To assess de-ubiquitination in the context of PRRSV-infected cells, HEK-293T cells were co-transfected with pcDNA3.1-Ub-HA and infectious cDNA clone of PRRSV-2 strain SD95-21 [either wild-type (WT) or mutant vKTF], from which PRRSV replication can be launched following the synthesis of full-length viral RNA from a CMV promoter [39]. At 48 h post-transfection, transfected cells were harvested using the lysis buffer described above. Cleared cell lysates were used for IP assays using an anti-GP5 pAb or anti-M pAb. Immunoprecipitated proteins were separated by SDS-PAGE, and GP5-Ub or M-Ub conjugates were detected by Western blot analysis with anti-HA mAb.

### Measurement of GP5 and M protein turnover

For the ectopic expression system, HEK-293T cells were transfected with a plasmid expressing GP5 or M protein, or co-transfected with plasmids expressing the nsp2TF and GP5 or M protein. For the purpose of comparison, HEK-293T cells were transfected with the empty vector only. At 24 h post-transfection, transfected cells were treated with the translation inhibitor cycloheximide (CHX, 30 μg/ml, Sigma) alone [40], or in combination with the proteasome inhibitor MG132 (20 μM). To further investigate GP5/M protein degradation in the context of viral infection, MARC-145 cells were infected with 0.5 moi of vKTF mutant or WT virus. At 24 h post infection (hpi), cells were treated with CHX (100 μg/ml) alone or with a combination of CHX and MG132 (20 μM). In both *in vitro* expression and infection conditions, whole cell lysates were collected at 0, 2, 4, 6 and 8 h post drug treatment, their total protein content was quantified, and 10 μg of each sample was subjected to Western blot analysis using a specific antibody to detect nsp2TF, GP5 or M protein expression. To measure the viral protein levels, protein bands on Western blots were selected using same-sized rectangles and their signal intensities were measured using the Analysis module of Image Studio Lite Ver 5.2 (Li-Cor). The relative abundance of protein species was calculated by dividing the intensity of the viral protein band by the intensity of the tubulin band.

### Statistical analysis

Graphpad Prism software (version 8) was used for statistical analysis and figure preparation. The one-way analysis of variance (ANOVA) was performed to evaluate statistical significance and a P value less than 0.05 was considered to be statistically significant.
